# Short-term effects of thinning on the understory natural environment of mixed broadleaf-conifer forest in Changbai Mountain area, Northeast China

**DOI:** 10.7717/peerj.7400

**Published:** 2019-07-26

**Authors:** Qiang Liu, Yue Sun, Gerong Wang, Fushan Cheng, Fucai Xia

**Affiliations:** 1 Forestry College, Beihua University, Jilin, Jilin, China; 2 Key Laboratory of State Forestry Administration on Conservation and Efficient Utilization of Characteristic Forest Resources of Changbai Mountain, Beihua University, Jilin, Jilin, China

**Keywords:** Forest microclimate, Soil environment, Intensity, Thinning

## Abstract

**Background:**

The understory natural environment is critical in affecting the succession and recovery process of vegetation, stand structure, and species composition of forest. The thinning intensity could significantly change the forest microclimates and soil properties, therefore, to analyze the effects of thinning intensity on the understory natural environment of forest is of important significance for promoting the ecological benefits of thinning.

**Methods:**

A total of 16 fixed sample plots with different thinning intensities were established in the mixed broadleaf-conifer forest in Jiaohe, situated in Changbai Mountain area, Northeast China, and the forest microclimates and soil properties were investigated after 4 years since the establishment of the sample plots.

**Results:**

The results showed that the high intensity thinning significantly decreased the leaf area index from 4.13 (unthinned plot) to 2.21 (high intensity thinned plot), and the air temperature was increased by thinning from May to July. Comparing with the unthinned plot, thinning caused a rise of temperature (ranging from 2.11 to 6.74 °C, depending on the intensity of thinning) in May. However, it showed cooling effect in September and October. Besides, the air moisture of thinning plots was lower than the control plot in May and October, when the density of leaves is lower in the forest, and it even decreased 20.27% after thinning. The thinning intensity had no significantly effect on water content and organic carbon in forest soils, and only the bulk density in the top-layer soils in high intensity thinning plot was remarkably increased. Total nitrogen in soil was increased by different intensities of thinning, and the availability of nutrients for nitrogen, phosphorus and potassium in some soils were also affected.

## Introduction

Thinning is an important and widely used silvicultural practice in forest ecosystem management. It reduces the tree density by cutting down and removing a proportion of trees in a forest with a relatively dense canopy, thereby redistributing the resources available for the remaining trees, improving nutrient availability, maintaining or improving the growth rate of the stand, and utilizing the intermediate source of timber revenue before the final forest is cut down at the end of the rotation (Juodvalkis, Kairiukstis & Vasiliauskas, 2005; Olivar et al., 2014; Rytter & Werner, 2007; Settineri et al., 2018; Tian et al., 2010). Thinning can assist in insect and fire prevention by developing a structurally and compositionally complex stand (Mcglone, Springer & Laughlin, 2009). Wildlife diversity may also increase in thinned forest plantations (Cahall, Hayes & Betts, 2013).

Environmental factors in the forest such as sunlight, water, temperature, and nutrients are critical in affecting the succession and recovery process of the vegetation, stand structure, and species composition of the forest (Breeuwer et al., 2009; Carvalho et al., 2017; Van Der Hoek, Van Mierlo Anita & Van Groenendael, 2010). Numerous studies have shown that illumination strength, temperature, moisture, and the content and availability of soil nutrients undergo corresponding changes after thinning (Bréda et al., 1995; Inagaki, Nakanishi & Fukata, 2011; Mattson & Smith, 1993; Moghaddas & Stephens, 2007; Shen et al., 2017). An increase in resource availability following thinning can lead to faster leaf-level rates of photosynthesis (Olivar et al., 2014) and also increases the light-use efficiency of retained trees depending on the availability of nutrients and water resources (West & Osier, 1995). The microclimates of forest light, temperature, and water are also important for understanding the ecological processes and functions determining the biophysical environmental conditions or resources (Ma et al., 2010; Spies & Franklin, 1989). Leaf area index (LAI) is an important parameter for characterizing the canopy structure of forest ecosystems and can also indirectly represent the light levels of the understory (Nagler et al., 2004; Smith, Sampson & Long, 1991).

Forest soil is the primary source of and storage system for available nutrients for plants, making it a key component in the ecosystem and providing an appropriate environment for tree growth. The silvicultural practice of thinning is expected to change soil environments by affecting the soil temperature and moisture, bulk density, microorganism conditions, quantity and quality of organic carbon in the soil, and by altering the soil’s nutrient dynamics (Barg & Edmonds, 1999). It is well known that soil bulk density (SBD) can affect plant growth by limiting the growth of plant shoots and roots (Houlbrooke et al., 1997; Materechera, Dexter & Alston, 1991; Tracy et al., 2013). Thus bulk density is an important factor for maintaining the community stability of plants in a mixed broadleaf-conifer forest, which could be significantly impacted by logging (Venanzi, Picchio & Piovesan, 2016). Organic carbon in the soil plays an important role in the terrestrial ecosystem carbon pool and has a profound effect on the physical, chemical, and biological properties of the soil (Benjamin, Mikha & Vigil, 2008; Eduardo et al., 2008; Hugar, 2017; Moskal et al., 2001; Motoki et al., 2014; Rawls et al., 2003). It is also one of the key indicators of soil fertility. The removal of organic matter will release the nutrients from logging residues by accelerating the decomposition and mineralization processes and will increase the amount of nutrients available for plants in both absolute and relative terms (Wollum & Schubert, 1975). Thinning may also reduce the nutrients returning to the forest soil and may result in an acceleration of soil weathering driven by biological processes (Blanco, Imbert & Castillo, 2008; Vadeboncoeur et al., 2014). It may also decrease carbon stocks in the forest soil and vegetation, thus increasing soil CO_2_ efflux at different degrees (Houghton, 2010; Mäkipää et al., 2015).

The natural mixed broadleaf-conifer forest is widely distributed throughout Europe, North America, and the Far East. It is also one of the main forest types in northeast China and makes an important contribution to the national economy for its large percentage of wood consumed for civil and national purposes (Chen et al., 2017; Inoue et al., 2017; Knoke et al., 2008; Li et al., 2014; Namikawa, Okamoto & Sano, 2000). The composition and structure of the forest type vary among regions. Thinning is one of the most important silvicultural measures performed in China to promote natural regeneration and maintain the continuity of the shelter benefits (Deng et al., 2014; He et al., 2015). However, previous studies have mainly focused on the effects of thinning on tree growth laws, stand volume, stand structure, and a single environmental condition (Cabon et al., 2018; Cheng, Yu & Wu, 2013; Roberts & Harrington, 2008; Huong, Mendham & Close, 2016; Nishizono, 2010). For example, Chen et al. (2017) revealed that thinning can optimize the spatial structure of trees and can also keep the stabilization of the spatial structure of the stand. There is still a lack of systematic study and discussion on how thinning might affect the forest microclimate and soil environment. Sustainable forest management has become a common point of awareness for foresters worldwide and in recent years the effect of thinning on the forest’s soil and microclimate and has become an important issue (Inagaki, Nakanishi & Fukata, 2011; Lee & Eo, 2018; Bach et al., 2010; Mäkipää et al., 2015; Vadeboncoeur et al., 2014). Understanding the changes in the temperature, moisture, and soil properties of the forest can help to improve the scientific management of the mixed broadleaf-conifer forest. The objectives of the proposed study are: (1) to compare the monthly changes in the microclimate conditions of the forest under four thinning intensities (0, 20, 40, and 60%); (2) to investigate the effects of thinning intensity on the soil properties of the understory to explore whether the thinning intensity will affect the natural environment of the understory, which is of significance for promoting the ecological benefits of thinning.

## Materials and Methods

### Study area

**Figure 1:**
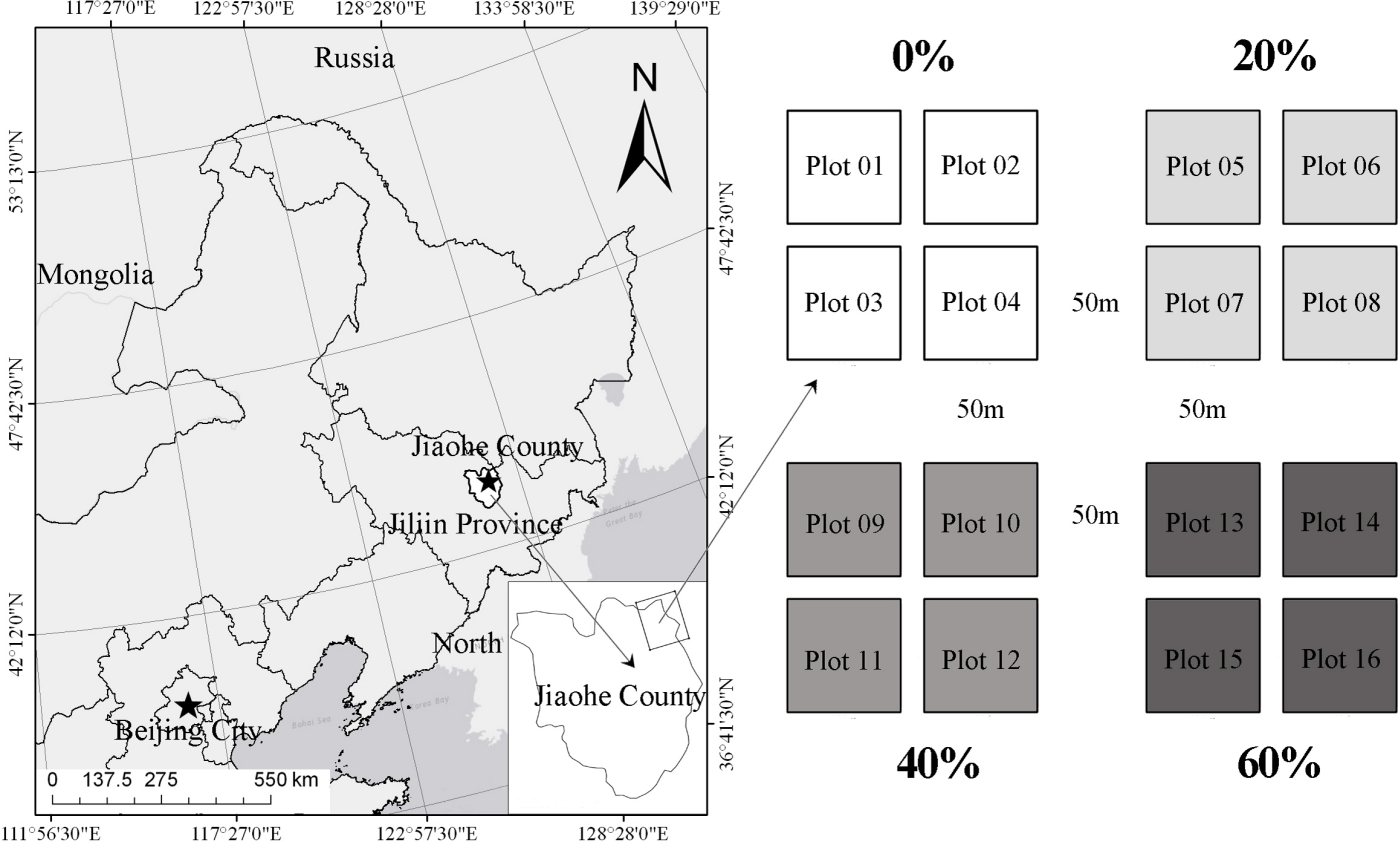
Location of sample plots.

The study was conducted in the Management Bureau of Jiaohe Forestry Experimental Area (127°35′–127°51′E, 43°51′–44°05′N) (Fig. 1), which is situated on Zhangguangcai Mountain of the Changbai mountain range with an operating area of 31,823 ha and an average altitude of 506 m. The climate is temperate continental monsoon, with four distinct seasons characterized by long, cold winters and generally short, warm summers. The mean annual temperature is 3.8 °C with a maximum average temperature of 21.7 °C in July and a minimum average temperature of −18.6 °C in January (Yao et al., 2016). The average depth of the upper limit of frozen soil is about 1.5–2.0 m. The annual precipitation is 700–800 mm with the heaviest rainfall occurring in June, July, and August. The major soil types include brown forest soil, swale soil, and herbal soil according to the Classification and Codes for Chinese Soil (National standard, GB/T 17296–2009), with brown forest soil covering 85% of the total area (Jiang, 2012). Vegetation is dominated by the flora of Changbai Mountain and the main forest type is a natural secondary forest of mixed broadleaf-conifer, with great richness and diversity in the tree species. Major arbor species in the study area include the *Pinus koraiensis* Sieb. et Zucc., *Ulmus japonica* Rehd., *Acer mono* Maxim., *Juglans mandshurica* Maxim., *Fraxinus mandshurica* Rupr., *Tilia amurensis* Rupr., *Acer mandshuricum* Maxim., *Quercus mongolica* Fisch. ex Ledeb., *Populus ussuriensis* Kom., *Ulmus laciniata* (Trautv.) Mayr., and *Maackia amurensis* Rupr. et Maxim. (Sun et al., 2017).

### Plot settings and soil sampling

In July 2011, 16 study plots (50 × 50 m) were established in the Dapuo forest farm within the study area (Fig. 1). A 10 m wide buffer zone on each side was included for each plot in order to reduce potential edge effects. A total of four treatments were conducted: (1) Plots 1–4: control group of intact forest (CON), (2) Plots 5–8: low-intensity thinning (20% of the trees removed) (LIT), (3) Plots 9–12: moderate-intensity thinning (40% of the trees removed) (MIT), (4) Plots 13–16: high-intensity thinning (60% of the trees removed) (HIT). The stand factors that were characteristic before and after thinning are summarized in Table 1.

**Table 1:** The stand factors characteristic before and after thinning.

Plot	Thinning intensity (%)	Location	Elevation (m)	Aspect	Slope (°)	Stand density (stems·hm^−2^)	Mean DBH (cm)	Mean height (m)	Basal area (m^2^)
						Before/After			
Plot 1–4	0	N: 43°57.9836′ E: 127°43.4723′	453	NE	1	1,106/1,106	14.6/14.6	9.7/9.7	30.1/30.1
Plot 5–8	20	N: 43°57.9625′ E: 127°43.5727′	443	NE	4	1,045/844	13.9/14.5	9.6/10.8	29.5/24.6
Plot 9–12	40	N: 43°57.9087′ E: 127°43.4510′	430	NE	5	1,007/770	14.8/14.8	9.7/9.9	30.4/20.4
Plot 13–16	60	N: 43°57.8784′ E: 127°43.5560′	447	NE	3	1,298/716	12.4/12.7	8.8/8.9	30.5/15.0

Each study plot was further divided into four subplots (25 × 25 m each) and two out of the four subplots within each plot were randomly selected for soil sampling. In May 2015, topsoil from 0 to 10 cm and 10 to 20 cm were sampled from the center of those selected subplots using the soil-drilling method. Approximately 200 g of soil from the surrounding areas of each site were also randomly sampled and mixed thoroughly to obtain a bulk sample. Soil samples were collected and stored in a polyethylene film bag after mixing for lab analysis. For the determination of the SBD, soil was collected using the cutting ring method and three random sampling points were selected as the repetitions around the central location of the subplots. Other parameters, including the air temperature and moisture in the forest and the LAI, were determined over 6 months from May to October. Four temperature and moisture meters were installed in each plot to monitor the air temperature and moisture. The LAI of each plot was determined using a Plant Canopy Analyzer (LAI-2200C), and it was measured three times each month, and data was collected randomly three to five times for each measurement. The LAI-2200C measured the gap fraction in five zenith angles with midpoints of 7°, 23°, 38°, 53°, and 67°, and calculated LAI based on Miller’s theorem (Chen et al., 2015). The locations of all sample sites were recorded using a hand-held global positioning system.

To pre-treat the soil samples, large fragments, such as gravel-sized materials and large plant roots, were removed from the samples and about 20 g of soil were taken for the measurement of moisture content. Soils were air dried at ambient temperature and were crushed to pass through a two mm stainless steel sieve. Portions of the crushed soils were further crushed to pass through one mm and 0.149 mm sieves, respectively and then stored in plastic bags prior to chemical analysis.

### Determination methods

Soil properties, including SBD, soil water content (SWC), soil organic carbon content (SOC), total and available nitrogen (TN and AN), total and available phosphorus (TP and AP), and total and available potassium (TK and AK) were determined, mostly following the standard methods published in the PRC Agricultural Industrial Standard. The SBD was determined using the cutting ring method (The Ministry of Agriculture of the People’s Republic of China, 2006). Specifically, the ring knife was inserted into the ground to obtain a soil core, which was then placed into a sterile plastic bag and sealed to preserve for lab drying. The weight of the drying soil was the SBD. The forest SWC was determined using the oven drying method (The Ministry of Agriculture of the People’s Republic of China, 1999), which was determined using the gravimetric method and expressed SWC as the mass ratio of water to dry weight; the constant weight was obtained after oven drying at 105 °C. The organic carbon in the forest soil was determined using the potassium dichromate oxidation and external heating method (The State Forestry Administration of the People’s Republic of China, 2015d; Nelson et al., 1982). The TN and AN in the forest soil were determined using the Kjeldahl method and the alkaline hydrolysis diffusion method, respectively (The State Forestry Administration of the People’s Republic of China, 2015a). AN was determined by a microdiffusion technique after alkaline hydrolysis. The TP and AP in the forest soil were determined with the alkali fusion method and acid soaking method, respectively (The State Forestry Administration of the People’s Republic of China, 2015b). TP was determined colorimetrically after wet digestion with H_2_SO_4_ + HClO_4_, and AP was extracted with 0.5 mol/l NaHCO_3_ solution (pH 8.5). Phosphate (P) in solution was determined colorimetrically by the formation of the blue phosphomolybdate complex following its reduction with ascorbic acid. The TK in the forest soil was determined using the alkali fusion method and the sample was melted by sodium hydroxide at a high temperature and then dissolved in water for determination by flame photometry and AK was determined by the CH_3_COONH_4_ extraction method (The State Forestry Administration of the People’s Republic of China, 2015c).

### Statistical analysis

One-way analysis of variance was used to compare the effects of different thinning intensities on the natural environment of the understory. The mean values of LAI, temperature, moisture, SBD, SWC, SOC, TN, AN, TP, AP, TK, AK that differed at *p* < 0.05 were considered significant trends. All the analyses were conducted using the SPSS 18.0 software.

## Results

### Effects of thinning intensity on forest microclimate

**Figure 2:**
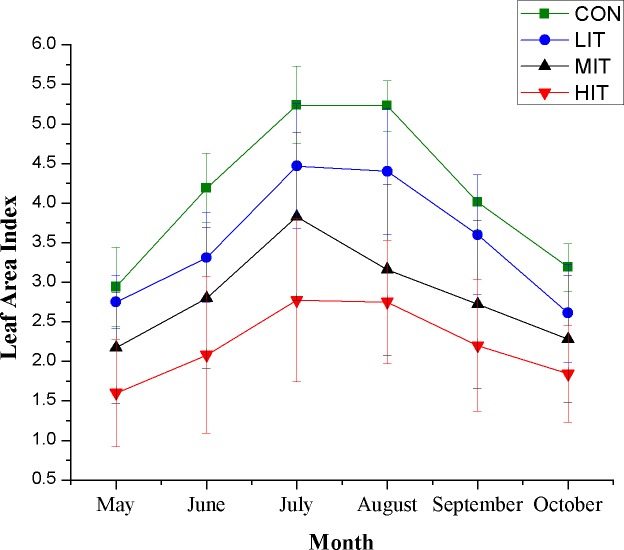
Monthly variation of leaf area index for different thinning plots. Meaning of abbreviations: CON, control group of intact forest; LIT, low-intensity thinning; MIT, moderate-intensity thinning; HIT, high-intensity thinning.

A monthly variation of LAI for thinning plots (Fig. 2) illustrates the effect of thinning intensity on the light condition of the understory. Over the growing season, the LAI for all plots revealed a similar trend, which increased from May to July and then decreased after August. Maximum LAI occurred between July and August. The thinning intensities greatly affected the LAI, as heavier thinning led to lower LAI (Table 2). Despite the slight difference between CON and LIT in May, the LAI varied significantly in other months and there were remarkable variations in different thinning plots. The monthly average LAI declined from 4.13 (CON) to 2.21 (HIT).

**Table 2:** The microclimate characteristics of different thinning plots.

Index	Month	CK	LT	MT	HT
Leaf area index	May	2.94 ± 0.50^a^	2.75 ± 0.34^a^	2.17 ± 0.70^b^	1.60 ± 0.68^c^
	June	4.19 ± 0.44^a^	3.31 ± 0.57^b^	2.80 ± 0.89^c^	2.08 ± 0.99^d^
	July	5.24 ± 0.49^a^	4.47 ± 0.79^b^	3.83 ± 1.06^c^	2.77 ± 1.03^d^
	August	5.23 ± 0.32^a^	4.40 ± 0.80^b^	3.16 ± 1.08^c^	2.75 ± 0.78^d^
	September	4.01 ± 0.35^a^	3.60 ± 0.76^b^	2.72 ± 1.06^c^	2.20 ± 0.83^d^
	October	3.19 ± 0.30^a^	2.61 ± 0.62^b^	2.28 ± 0.80^c^	1.84 ± 0.61^d^
Air temperature (°C)	May	10.07 ± 4.03^a^	12.18 ± 5.08^b^	16.81 ± 6.09^c^	13.32 ± 5.16^d^
	June	16.39 ± 5.01^a^	15.83 ± 3.18^a^	16.45 ± 2.70^a^	18.53 ± 2.99^b^
	July	19.28 ± 4.16^a^	20.39 ± 3.04^b^	20.32 ± 2.75^b^	20.76 ± 2.70^b^
	August	22.02 ± 3.66^a^	21.6 ± 3.29^ab^	21.12 ± 3.56^b^	21.53 ± 1.51^ab^
	September	16.13 ± 4.56^a^	14.13 ± 5.41^b^	15.00 ± 4.01^b^	13.39 ± 4.79^c^
	October	6.21 ± 6.49^a^	5.36 ± 6.10^b^	4.65 ± 6.02^b^	2.97 ± 4.41^c^
Air moisture (%)	May	84.78 ± 19.62^a^	72.37 ± 24.15^b^	65.61 ± 24.53^c^	79.37 ± 20.18^d^
	June	78.11 ± 15.93^a^	90.30 ± 13.28^b^	97.88 ± 3.78^c^	90.80 ± 11.30^b^
	July	88.71 ± 11.55^a^	93.11 ± 8.90^b^	94.45 ± 5.97^b^	98.95 ± 2.23^c^
	August	98.51 ± 3.56^a^	95.72 ± 6.73^b^	88.38 ± 10.24^c^	74.78 ± 9.45^d^
	September	83.24 ± 10.94^ab^	83.91 ± 14.78^ab^	86.41 ± 16.89^a^	82.28 ± 19.56^b^
	October	78.40 ± 18.19^a^	70.52 ± 22.61^b^	58.13 ± 20.49^c^	63.31 ± 17.72^d^

**Note:**

The values with different lowercase superscripts mean significant difference among four thinning treatments (*p* < 0.05).

**Figure 3:**
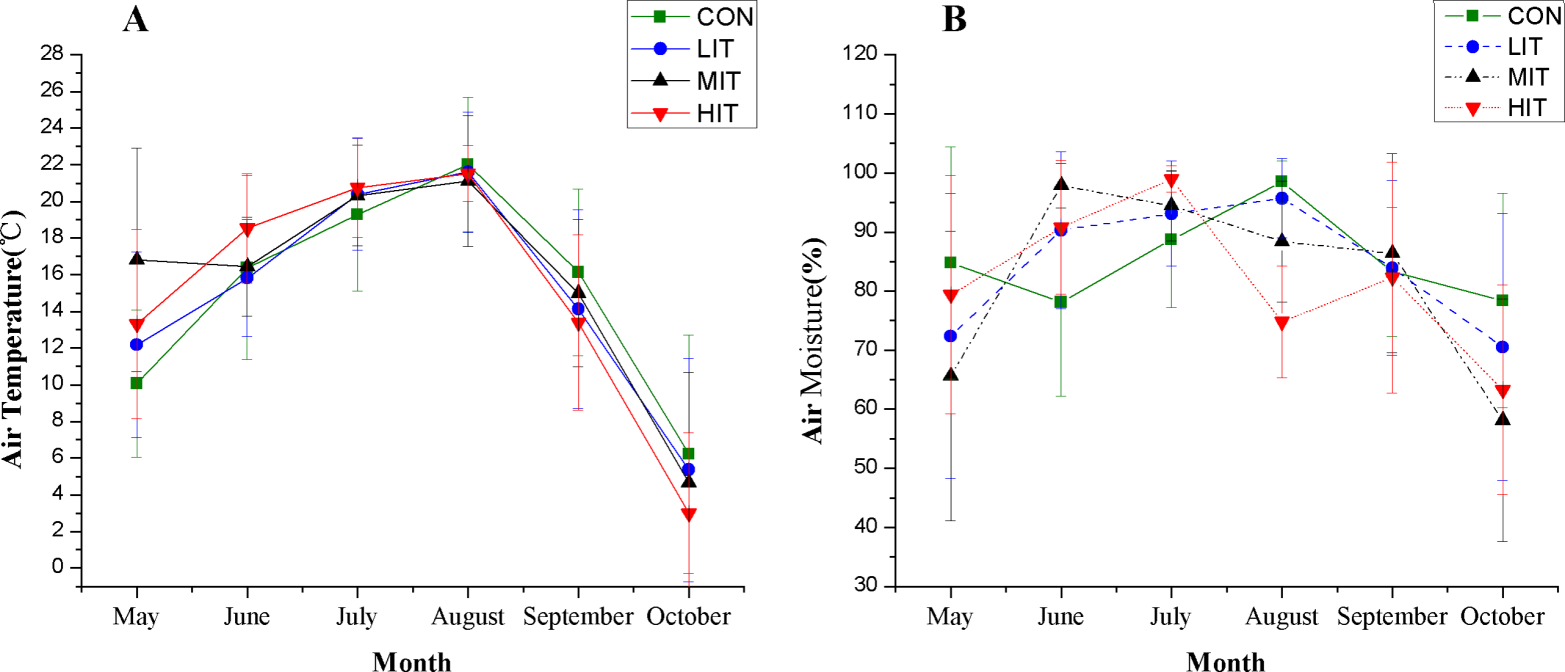
Understory temperature (A) and moisture (B) in different thinning plots. Meaning of abbreviations: CON, control group of intact forest; LIT, low-intensity thinning; MIT, moderate-intensity thinning; HIT, high-intensity thinning.

The air temperature in the investigated mixed broadleaf-conifer stand increased at first and then decreased from May to October (Fig. 3A; Table 2) and all thinning plots showed higher (2.11–6.74 of increase) air temperatures than CON in May. However, there was no significant difference between LIT, MIT, and CON in June, while HIT still had a higher air temperature. The air moisture of the mixed broadleaf-conifer forest varied in different months as the rainfall changed and thinning also had an obvious effect on air moisture (Fig. 3B; Table 2). In May, August, and October the air moisture of the CON plot was significantly higher than the others and the plots with moderate and high intensity thinning maintained relatively lower air moisture, even seeing it decrease by 20.27% in the MIT plot in October. However, for June, July, and September, the thinning plots showed higher air humidity compared to CON and the plots with higher thinning grades were more humid. The MIT plot showed higher moisture in June and increased air moisture relative to HIT, with a significant difference.

### Effects of thinning intensity on soil properties

#### Effects of thinning intensity on SBD, SWC, and SOC

**Figure 4:**
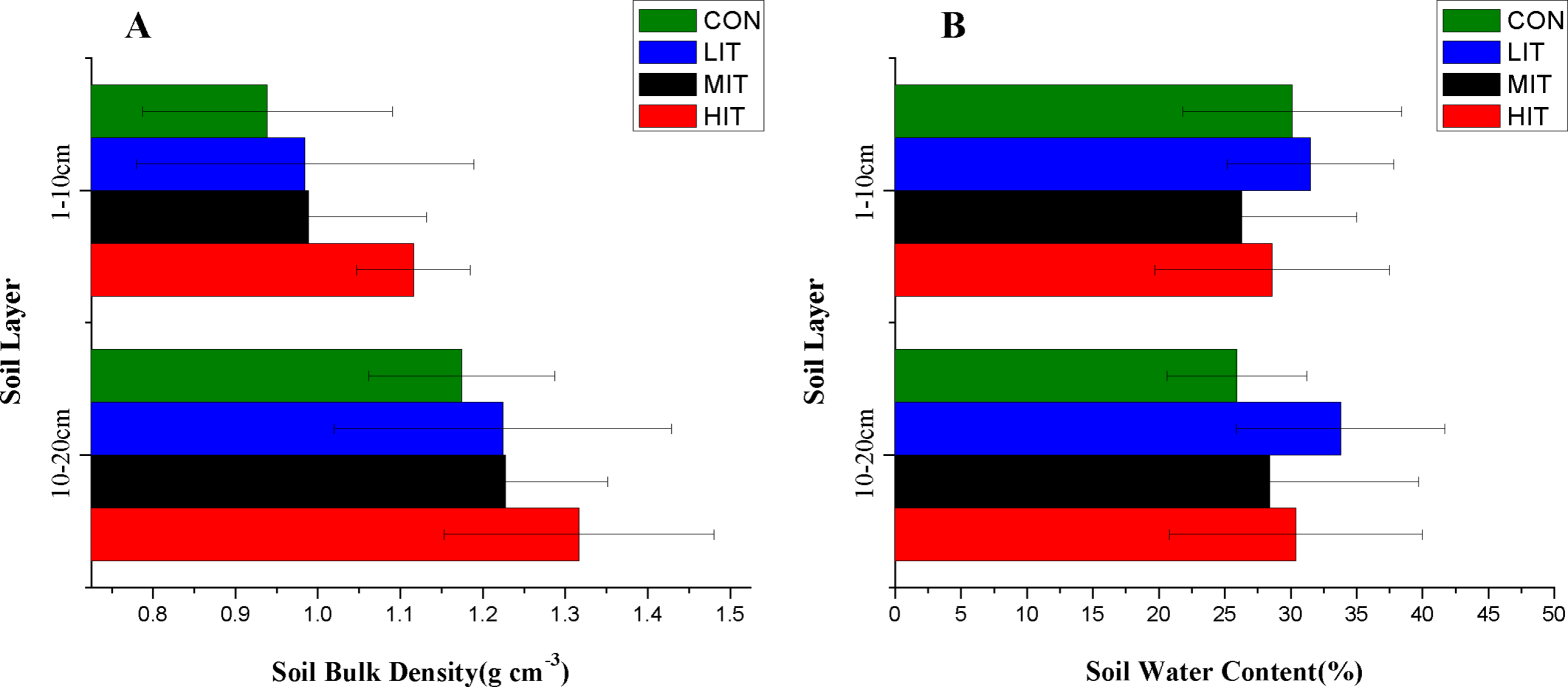
Bulk density (A) and water content (B) of forest soils in different thinning plots. Meaning of abbreviations: CON, control group of intact forest; LIT, low-intensity thinning; MIT, moderate-intensity thinning; HIT, high-intensity thinning.

**Figure 5:**
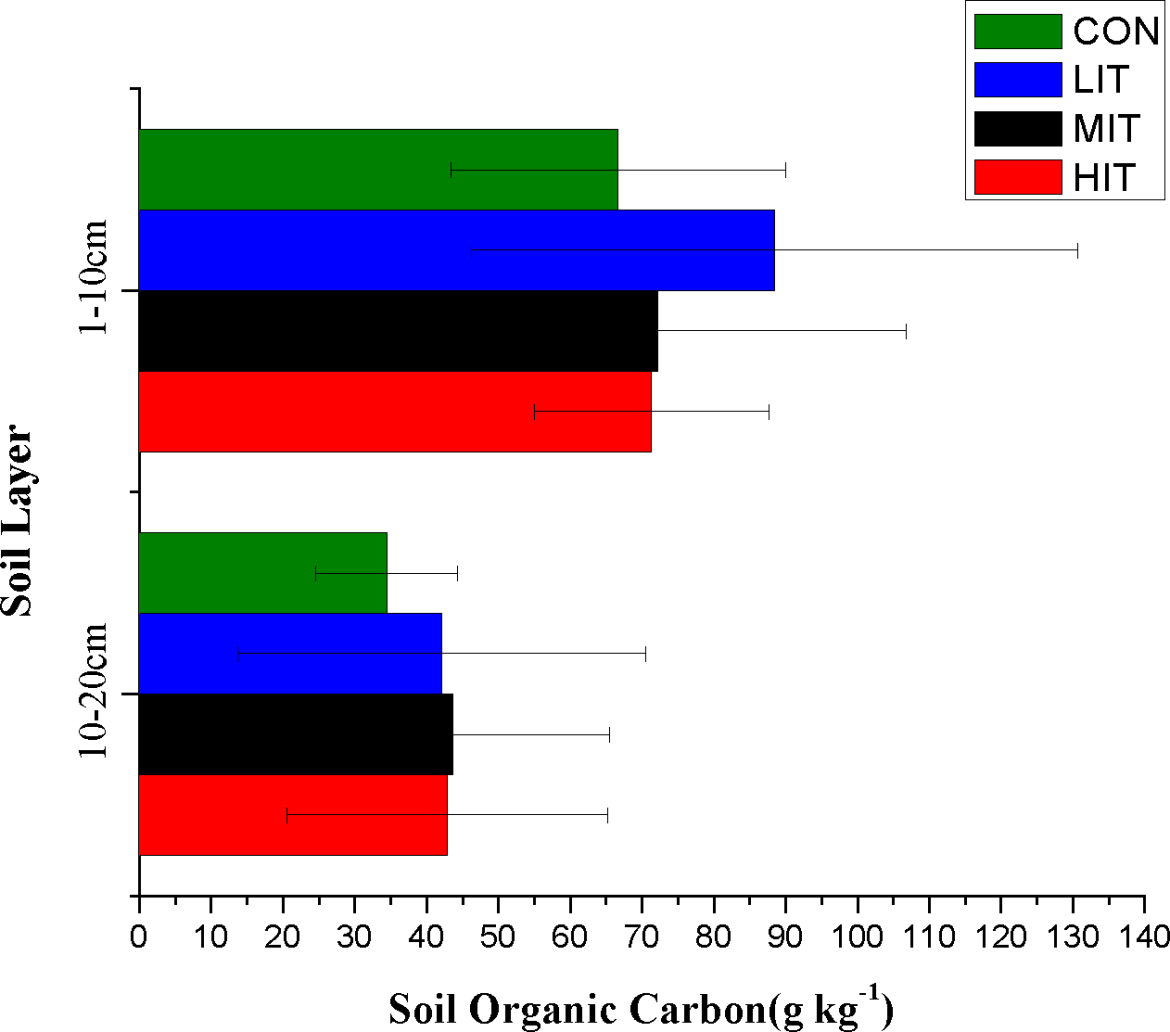
Organic carbon content of forest soils in different thinning plots. Meaning of abbreviations: CON, control group of intact forest; LIT, low-intensity thinning; MIT, moderate-intensity thinning; HIT, high-intensity thinning.

In the current study, the mean values of SBD in 0–10 cm topsoil varied from 0.9386 to 1.1158 g cm^−3^ (Fig. 4A; Table 3) and the SBD in HIT soils was significantly higher than CON (*p* < 0.05). However, there were no statistically significant differences between various thinning intensities for the 10–20 cm soil layer, indicating that a higher intensity of thinning will notably influence the bulk density of 0–10 cm topsoil but have no obvious influences on deeper soil. No significant difference of SBD was observed between the 0 and 10 cm and 10 and 20 cm soils 4 years after thinning. The average water content of soils in different thinning plots varied from MIT (26.3%) to LIT (31.5%) for the 0–10 cm layer, and from CON (25.9%) to LIT (33.8%) for the 10–20 cm layer, respectively (Fig. 4B; Table 3). The results showed that thinning intensities made no significant difference among the different thinning intensities and soil depths for SWC. Figure 5 shows the effect of thinning on the content of SOC in different soil layers The mean values of SOC in the 0–10 cm soil varied from 34.46 g kg^−1^ for LIT to 88.46 g kg^−1^ for MIT, and from 42.89 g kg^−1^ for HIT to 72.14 g kg^−1^ for CON. There were no significant variations of SOC in the plots with different thinning intensities but the top-layer soils showed significantly higher SOC than the deep layer (Table 3).

**Table 3:** The physical and chemical characteristics of different thinning plots.

Indexes	Intensity							
	CON		LIT		MIT		HIT	
	0–10 cm	10–20 cm	0–10 cm	10–20 cm	0–10 cm	10–20 cm	0–10 cm	10–20 cm
SBD (g cm^−3^)	0.9386 ± 0.15^aA^	1.1744 ± 0.11^aA^	0.9843 ± 0.20^aAB^	1.2243 ± 0.20^aA^	0.9881 ± 0.14^aAB^	1.2271 ± 0.12^aA^	1.1158 ± 0.07^aB^	1.3168 ± 0.16^aA^
SWC (%)	30.1 ± 8.3^aA^	25.9 ± 5.3^aA^	31.5 ± 6.3^aA^	33.8 ± 7.9^aA^	26.3 ± 8.7^aA^	28.4 ± 11.3^aA^	28.6 ± 8.9^aA^	30.4 ± 9.6^aA^
SOC (g kg^−1^)	66.66 ± 23.30^aA^	34.46 ± 9.89^bA^	88.46 ± 42.2^aA^	42.15 ± 28.42^bA^	72.14 ± 34.59^aA^	43.72 ± 21.81^bA^	71.29 ± 16.32^aA^	42.89 ± 22.30^bA^
TN (g kg^−1^)	3.32 ± 0.84^aA^	1.91 ± 0.52^bA^	6.72 ± 2.66^aB^	3.99 ± 1.87^bB^	5.64 ± 2.05^aAB^	3.32 ± 1.73^bB^	5.98 ± 1.26^aB^	3.84 ± 1.68^bB^
AN (mg kg^−1^)	221.51 ± 114.82^aA^	132.20 ± 14.25^bA^	335.84 ± 142.11^aB^	246.68 ± 122.45^aB^	276.05 ± 98.97^aAB^	164.69 ± 93.25^bA^	218.31 ± 104.23^aA^	135.47 ± 61.57^bA^
TP (g kg^−1^)	0.313 ± 0.075^aA^	0.209 ± 0.051^bA^	0.376 ± 0.216^aA^	0.230 ± 0.075^aA^	0.194 ± 0.068^aA^	0.210 ± 0.118^aA^	0.305 ± 0.103^aA^	0.294 ± 0.105^aA^
AP (mg kg^−1^)	14.88 ± 6.39^aA^	6.44 ± 1.54^bA^	31.05 ± 8.99^aB^	9.25 ± 2.93^bA^	30.16 ± 13.13^aB^	17.08 ± 11.29^bB^	19.41 ± 6.45^aA^	11.95 ± 6.51^bAB^
TK (g kg^−1^)	4.10 ± 0.09^aA^	4.12 ± 0.08^aA^	4.05 ± 0.08^aA^	4.09 ± 0.09^aA^	4.06 ± 0.31^aA^	4.08 ± 0.37^aA^	4.19 ± 0.13^aA^	4.19 ± 0.21^aA^
AK (mg kg^−1^)	74.87 ± 6.14^aA^	62.23 ± 9.42^bA^	77.75 ± 8.72^aA^	56.37 ± 12.33^bA^	84.98 ± 5.65^aB^	75.34 ± 7.54^bB^	87.05 ± 6.42^aB^	72.71 ± 9.36^bB^

**Note:**

The values with different lowercase superscripts mean significant difference between two soil layers under the same thinning intensity (*p* < 0.05), values with different capital letter superscripts mean significant difference among four thinning treatments in the same soil layer (*p* < 0.05).

### Effects of thinning intensity on nutrients in soil

**Figure 6:**
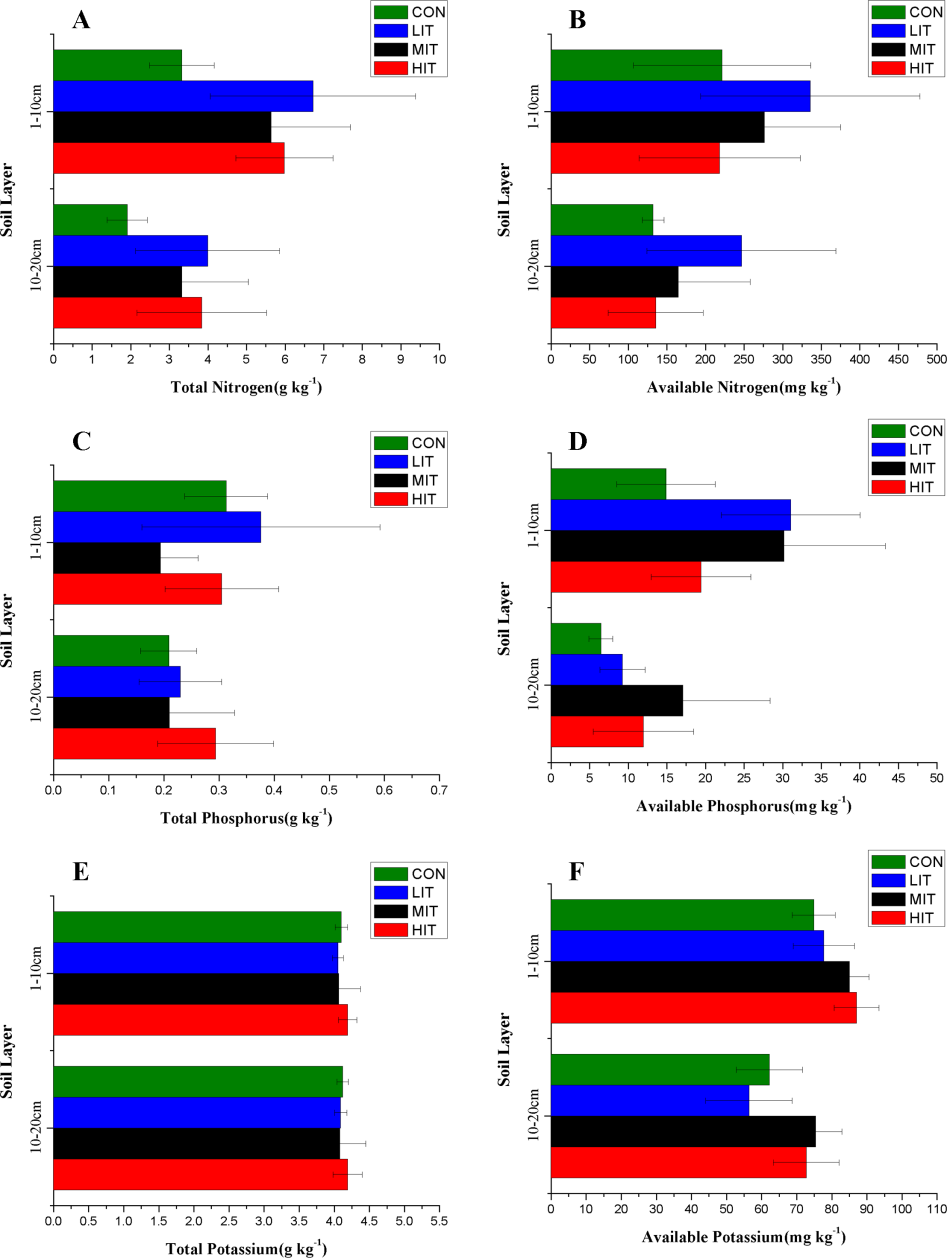
Contents of TN (A), AN (B), TP (C), AP (D), TK (E), and AK (F) in forest soils of different thinning plots. Meaning of abbreviations: CON, control group of intact forest; LIT, low-intensity thinning; MIT, moderate-intensity thinning; HIT, high-intensity thinning.

The concentrations of TN, TP, TK, AN, AP, and AK in different thinning plots were presented in Fig. 6 and Table 3. The variations of TN concentrations in both the 0–10 cm and 10–20 cm soil layers followed: LIT > HIT > MIT > CON (Fig. 6A). The AN contents in the top layer followed: LIT > MIT > CON > HIT, and LIT > MIT > HIT > CON for the deep layer (Fig. 6B). Unlike the TN in forest soils, no significant differences were observed in the TP and TK content under different thinning grades and soil layers. The TP content in soils were in the order of LIT > CON > HIT > MIT for the top layer and HIT > LIT > MIT > CON for the deep layer (Fig. 6C), respectively. Thinning caused different amounts of increase of AP content in different plots and LIT and MIT were significantly higher than CON and HIT in the top layer, however, MIT had the highest concentration of AP for the deep layer and MIT and HIT were significantly higher than CON and LIT (Fig. 6D). Thinning intensities had no significant effect on the TK contents but the mean AK contents in the MIT and HIT plots were higher than CON and LIT for both the top and deep layers, respectively (Figs. 6E and 6F), indicating that high intensity thinning had a significant positive effect on promoting AK concentrations.

## Discussion

Forest thinning could not only improve the remaining tree growth and stand regeneration by redistributing resources and improving nutrient availability (Tian et al., 2010), but also change the forest microclimate. The current study confirmed that thinning significantly affects the microclimate, which is in agreement with numerous other studies (Heithecker & Halpern, 2006; Ma et al., 2010; Tang et al., 2005). Thinning decreases the canopy cover of the forest directly by cutting down and removing a proportion of the trees, resulting in more open areas (Hale, 2003) such that more direct sunlight reaches the forest floor and heats up the atmosphere near the ground. Therefore, the transmittance is significantly different in thinned vs. unthinned forests, which is indicated by the reduction of LAI in the current study, and thinning significantly improved the lighting condition of the understory. The result is consistent with the conclusions of previous research on the variation of LAI after thinning in forest ecosystems (Cutini, 1996; Ma et al., 2010; Sano et al., 2012).

The changes in radiation also have cascading effects on temperature, moisture, and energy balance since solar radiation provides the primary energy to the ecosystem (Aussenac, 2000). Thus, the trend of air temperature in the investigated mixed broadleaf-conifer stand had the same seasonal variation to LAI for different thinning plots. July and August are the two hottest months in the year and the air temperature in thinned plots was significantly higher than CON due to the fact that the CON plot had stronger shade effects caused by a relatively high stand density. In August, only the MIT plot maintained a relatively low temperature when compared with CON. In September and October, the air temperature of the thinned plots was much lower than CON and the temperature varied among plots, mostly because the absorption capacity for ground radiation had been changed dramatically since thinning affected the forest canopy structure (Sader, 1986; Seyednasrollah, Kumar & Link, 2013). The air moisture of the forest also varied among different thinning intensities. Our results showed a drying effect on forest air during the drier season, and the same conclusion is reported in Rambo & North (2009). We also found that the moisture of the CON plot was significantly higher than the other thinned plots in this study, and that the plots with higher thinning grades maintained relatively lower air moisture.

Our study also confirmed that soil properties are greatly affected by thinning. As one of the important factors affecting the soil water distribution, SBD affects the water storage capacity of soil in the way that the increase of SDB results in a decrease in the water storage capacity (Bangitaa & Rajashekhar Rao, 2012; Chen et al., 2014). The current study results showed that high intensity thinning could significantly increase the SBD for the top soil but had no obvious influence on deeper soil. In the vertical direction, there was no significant difference in SBD between 0–10 cm and 10–20 cm soils 4 years after thinning. The deeper soil had higher SBD, and a similar study on the pine-oak mixed forest in the Qinling mountains of China showed that as time progressed, the bulk density would significantly increase as the soil became deeper (Chen et al., 2014). There was also no significant change in SWC before and after thinning. In fact, a long-term study done by Gray, Spies & Easter (2002) showed an absence of differences in SWC between thinned and unthinned treatments after several years due to the development of vegetation in the thinned stands. Although thinning intensities significantly increase the decomposition of organic matter as a result of an increase in microbial activity (Tian et al., 2010), no significant changes in SOC were found for different plots in our study in the short term thinning effects. Furthermore, comparison of the vertical distributions of SOC for the mixed broadleaf-conifer forest in each plot showed larger differences (*p* < 0.05) between the top layer and deep layer and the result was in accordance with experimental results from other relevant studies (Tian et al., 2010; Mingfang, Lijun & Weida, 2012; Yuan et al., 2010). On average, the SOC content in 10–20 cm soil was only about 50% of 0–10 cm in the current study.

The TN and AN content decreased from the top to the deep layers and showed an upper-accumulated pattern, except for AN in the LIT plot. However, the concentrations of TN and AN were significantly higher in the LIT plot than that in the CON. The thinning intensities significantly increased TN in the deep-layer soils and the TN in the top soils were also higher than CON in the thinning plots. However, the MIT and HIT had little effect on the AN content of the forest soils since nitrogen in the forest floor is resistant to breakdown and a large proportion of the decomposed nitrogen is subjected to biological fixation in situ (Wright, 1957). Although the TP contents were relatively stable after thinning, the AP accumulated in the surface soil to a greater extent in the LIT than in the deeper layer, indicating that low intensity thinning played a positive role in the mineralization or activation of phosphorus in the surface soil. Previous studies indicated that more readily available forms of phosphorus would be plentiful after thinning compared to CON (Smolander et al., 2013) This may be caused by the high content of the organic fraction of soil phosphorus in CON, which undergoes a slow process of mineralization and is relatively unavailable to plants (Phiri et al., 2001). Moreover, thinning had no significant effect on the TK contents in soils, while higher intensities of thinning had a greater positive effect on promoting AK concentrations.

By deriving relationships from commonly collected field data, the effects of thinning on forest microclimates and soil properties could be estimated before any treatment is applied. Such information could be useful for forest managers who could then take potential ecological consequences and the tradeoffs of management activity into account before actually applying treatments (Heithecker & Halpern, 2006).

## Conclusions

The study of the effects of thinning on the forest microclimate and soil properties showed that thinning intensities had different effects on the natural environment of the understory of a mixed broadleaf-conifer forest. Thinning significantly and directly changed the stand density and thereby altered the LAI. High intensity thinning greatly decreased the LAI and improved the lighting condition of the understory. Over the growing season, high intensity thinning significantly increased the air temperature from May to July, while in September and October it showed a greater cooling effect. The air moisture of the mixed broadleaf-conifer forest also varied monthly under different intensities of thinning. The moisture of the plots without thinning were higher than the thinned plots in May and October, when there is a lower leaf density in the forest and thinning could significantly increase the moisture in June and July. Thinning also changed the soil properties, especially the soil nutrient dynamics, but it did not significantly affect SWC and SOC in forest soils. Only the bulk density in the top-layer of soil in the high intensity thinning plot was remarkably increased comparing to the unthinned stand. The soil nutrient, TN, was increased after different intensities of thinning and the available nutrients of nitrogen, phosphorus, and potassium in some soils were affected by thinning intensities to varying degrees. The nutrient bioavailability could be enhanced after certain intensities of thinning. Overall, this study demonstrated that the thinning intensity had short-term effects on the natural environment of the forest and provides important theoretical guidance and experimental data for forest management.

## Supplemental Information

10.7717/peerj.7400/supp-1 Supplemental Information 1 Raw data of the changes of forest microclimates and soil environment in the investigated mixed broadleaf-conifer stand after different intensities thinning.

## Additional Information and Declarations

### Data Availability

The following information was supplied regarding data availability: The raw measurements are available as a Supplemental File. The raw data shows all changes of forest microclimate and soil environment in the investigated mixed broadleaf-conifer stand after thinning with different intensity.
